# Étude analytique et descriptive de la survie des patients atteints de métastases vertébrales en milieu rhumatologique au Togo

**DOI:** 10.11604/pamj.2026.53.90.49285

**Published:** 2026-02-19

**Authors:** Sadat Oniankitan, Mamadou Lamine Diallo, Eyram Fianyo, Awaki Esso Ataké, Mamadou Baïlo Diallo, Tagbor Cyril Komi, Viwale Etonam Sika Koffi-Tessio, Kodjo Kakpovi, Prenam Houzou, Owonayo Oniankitan

**Affiliations:** 1 Service de Rhumatologie, Centre Hospitalier Universitaire de Kara, Kara, Togo,; 2 Service de Rhumatologie, Centre Hospitalier Universitaire Ignace Deen, Conakry, Guinée,; 3 Service de Rhumatologie, Centre Hospitalier Régional de Lomé Commune, Lomé, Togo,; 4 Service de Rhumatologie, Centre Hospitalier Universitaire de Syvanus Olympio, Lomé, Togo,; 5 Disease Prevention and Control Department, District Heath Management Board, Conakry, Guinea,; 6 Service de Rhumatologie, Hôpital de Bè, Kara, Togo,; 7 Service de Rhumatologie, Centre Hospitalier Régional de Tsévié, Lomé, Togo,; 8 Service de Rhumatologie, Centre Hospitalier Régional de Kara, Kara, Togo

**Keywords:** Métastases, pronostic, survie, Tokuhashi, Togo, Metastases, prognosis, survival, Tokuhashi, Togo

## Abstract

Les métastases, surtout osseuses, sont fréquentes au cours de l'évolution de certains cancers et engagent le plus souvent le pronostic vital, voire le pronostic fonctionnel (compression médullaire) des patients. L'objectif de cette étude est d'évaluer la survie des patients souffrant de métastases vertébrales au Togo. Nous avons mené une étude rétrospective de type transversal, descriptive et analytique de 5 ans, de 2016 à 2020 dans les services de rhumatologie des centres hospitaliers universitaires (CHU) Kara, Sylvanus Olympio et de l'hôpital de Bè du Togo, chez tous les patients diagnostiqués de métastases osseuses et/ou métastases viscérales d'une tumeur primitive. Le score de Tokuhashi a été utilisé. Une analyse de régression multivariée a été faite afin de déterminer les facteurs associés. Nous avons colligé 82 patients avec une prédominance masculine (53 hommes, soit 64,63%), d'âge moyen de 65 ans chez les hommes. Le cancer de la prostate était retrouvé chez 46 patients (56,09%), suivi du cancer du sein chez 12 patients (14,63%). Les facteurs statistiquement associés au mauvais pronostic étaient la présence d'une image radiographique lytique (ORa: 3,87; IC à 95%: 1,66 - 4,85) et d'une image mixte (ORa: 2,58; IC à 95%: 1,38 - 4,74) avec respectivement p<0,004 et p<0,001. Dans notre étude, 63,3% des patients qui avaient un mauvais pronostic ont survécu moins de 6 mois (ORa: 4,99; IC à 95%: 7,26 - 40,0) avec p = 0,001. Une analyse multivariée entre les tumeurs les plus fréquentes et la survie selon le score de Tokuhashi a retrouvé que le cancer de la prostate (n = 46) était associé à une survie médiane réelle de 17,9 mois (Ora: 0,23; IC à 95%: 0,11-0,48); contre une survie prédite par le score de Tokuhashi de 9,87 mois. Le cancer du sein (n = 12) montrait une survie réelle de 9,87 mois (ORa: 0,48; IC à 95%: 0,21-1,14) avec une survie prédite de 8,92 mois. Les métastases osseuses sont associées à une morbidité importante et à la survie des patients. La présence d'une image radiographique lytique et d'une image mixte est le facteur statistiquement significatif de mauvais pronostic. Le cancer de la prostate et celui du sein sont les cancers les plus fréquents et statistiquement associés à la survie des patients selon le score de Tokuhashi, avec une survie réelle de 17,9 mois contre une survie prédite par le score de Tokuhashi de 9,87 mois et une survie réelle de 9,87 mois contre une survie prédite de 8,82 mois.

## Abstract (en)

Metastases, especially bone metastases, are common during the progression of certain cancers and most often threaten the patient's life or sometimes their functional prognosis (spinal cord compression). The objective of this study is to evaluate the survival of patients with vertebral metastases in Togo. We conducted a retrospective, cross-sectional, descriptive, and analytical study over 5 years, from 2016 to 2020, in the Rheumatology Departments of Kara University Hospital, Sylvanus Olympio, and Bè Hospital in Togo, including all patients diagnosed with bone and/or visceral metastases from a primary tumor. The Tokuhashi score was used. Multivariate regression analysis was performed to identify associated factors. We collected data on 82 patients, predominantly male (53 men, 64.63%), with a mean age of 65 years for the men. Prostate cancer was found in 46 patients (56.09%), followed by breast cancer in 12 patients (14.63%). Factors statistically associated with poor prognosis were the presence of a radiographic lytic image (ORa: 3.87; 95% CI: 1.66 - 4.85) and a mixed image (ORa: 2.58; 95% CI: 1.38 - 4.74) with p<0.004 and p<0.001, respectively. In our study, 63.3% of patients with a poor prognosis survived less than 6 months (ORa: 4.99; 95% CI: 7.26 - 40.0) with p = 0.001. A multivariate analysis between the most frequent tumors and survival according to the Tokuhashi score found that prostate cancer (n = 46) was associated with a median actual survival of 17.9 months (OR: 0.23; 95% CI: 0.11-0.48), versus a Tokuhashi score predicted survival of 9.87 months. Breast cancer (n = 12) showed an actual survival of 9.87 months (OR: 0.48; 95% CI: 0.21-1.14) with a predicted survival of 8.92 months. Bone metastases are associated with significant morbidity and patient survival. The presence of a lytic radiographic image and a mixed image are statistically significant factors of poor prognosis. Prostate cancer and breast cancer are the most frequent cancers and are statistically associated with patient survival according to the Tokuhashi score, with an actual survival of 17.9 months against a Tokuhashi score-predicted survival of 9.87 months, and breast cancer with an actual survival of 9.87 months against a predicted survival of 8.82.

## Introduction

L'apparition de métastases, dont la localisation osseuse, surtout vertébrale, est fréquente au cours de l'évolution clinique de nombreux cancers, constitue un drame de santé publique pouvant engager à la fois le pronostic fonctionnel et vital [1,2].

En Afrique subsaharienne, plusieurs études hospitalières ont été faites sur les métastases osseuses et ces études sont surtout d'ordre épidémiologique, sémiologique [3-5]. De nos jours, il existe plusieurs outils ou scores permettant d'évaluer le pronostic vital et la survie des métastases osseuses. Ces scores pronostiques disponibles sont entre autres le score de Tomita, le score de Tokuhashi modifié et le score de Katagri, dont les deux premiers sont les plus utilisés de nos jours [6-8]. Malgré ces scores, les études sur le pronostic vital des métastases osseuses sont rares dans notre contexte.

Vu la fréquence élevée des métastases osseuses dans notre contexte, l'absence d'études antérieures sur le pronostic de ces tumeurs, le coût élevé des examens d'imagerie existants, l'absence de dosage de certains marqueurs tumoraux, l'absence de scintigraphie osseuse et de TEP scanner sont les principaux motifs de réalisation de cette étude dans le but d'harmoniser un suivi correct selon l'évolution de la tumeur. L'objectif général de cette étude était d'évaluer la survie des patients souffrant de métastases vertébrales en milieu rhumatologique togolais.

## Méthodes

**Conception et contexte de l'étude:** nous avons réalisé une étude rétrospective de type transversal, descriptive et analytique dans les services de rhumatologie du Togo, à savoir les CHU Kara et Sylvanus Olympio et l'Hôpital de Bè. Il s'agit des 3 principaux services de rhumatologie du pays.

**Durée de l'étude:** cette étude a été réalisée de janvier 2016 à décembre 2020, soit une durée de 5 ans.

**Population étudiée:** l'étude a concerné tous les patients chez qui le diagnostic de tumeur osseuse primitive ou secondaire (quelle que soit la localisation) d'une quelconque tumeur primitive pendant la période d'étude.

**Critère d'inclusion:** tous les patients présentant des métastases osseuses (quelle que soit la localisation) et/ou viscérales d'une tumeur primitive quelconque.

**Critère d'exclusion:** les patients ayant souffert d’une tumeur osseuse maligne primitive ou d’une tumeur osseuse bénigne n'ont pas été inclus dans l'étude.

**Procédure d'échantillonnage:** nous avons procédé à un échantillonnage consécutif des dossiers des patients ayant souffert de métastases osseuses d'une tumeur primitive pendant la période d'étude.

**Recueil des données:** pour la collecte des données, nous avons utilisé comme supports les registres d'hospitalisation pour déterminer la période d'hospitalisation, les dossiers des patients pour les renseignements sémiologiques (diagnostiques de métastases osseuses). Nous avons contacté par appel téléphonique les proches des patients non revus pour le suivi pendant la période d'étude ou pour déterminer la période de décès.

### Les variables étudiées

**Variables épidémiologiques:** elles correspondaient aux variables socio-démographiques telles que l'âge et le sexe du patient.

**Variables sémiologiques:** elles étaient représentées par les motifs de consultation qui sont dominés par les rachialgies avec impotence fonctionnelle, l'altération de l'état général (définie dans le score de performance de Karnofsky), la durée d'évolution des symptômes (le délai qui s'est écoulé entre la survenue des symptômes et son hospitalisation. Cette durée a été évaluée en mois dans notre étude), les antécédents et/ou les comorbidités du patient. Pour déterminer la tumeur primitive, l'étiologie a été posée par présomption du fait de l'absence de preuves histologiques (biopsie). Ainsi, les signes fonctionnels, les marqueurs tumoraux spécifiques de certaines tumeurs, les caractéristiques radiologiques (ostéocondensation, ostéolyse et rarement mixte) ont été utilisés pour déterminer l'étiologie primitive. a) score de Tokuhashi (utilisé pour déterminer la probabilité de survie du patient); b) variables évolutives (date de décès du patient que nous avons pu renseigner grâce aux suivis et aux appels téléphoniques des proches des patients). Le score de Tokuhashi a été utilisé dans notre étude, à cause de sa facilité et même pour certains auteurs il ne l'est pas [7]. Le score de Tokuhashi modifié est un score clinico-imagérique composé de 5 éléments: a) l'état général du patient défini par l'indice de performance de Karnofsky, qui identifie trois stades un bon état général (indice de performance entre 80 à 100%), un état général modéré (indice de performance entre 50 à 70%) et un mauvais état général (indice de performance entre 10 à 40%); b) l'atteinte neurologique défini par le score pronostic de Fankel, qui identifie 5 grades (grade A: paralysie complète; grade B: paralysie motrice complète avec conservation d'une sensibilité; grade C: paralysie motrice incomplète ne permettant pas la déambulation; garde D: paralysie motrice partielle autorisant la marche, atteinte sensitive; grade E: absence d'atteinte neurologique) [9]; c) la tumeur primitive; d) le nombre de métastases vertébrale; e) le nombre de métastases extra vertébrale.

Le Tableau 1 présente le score pronostique de Tokuhashi [7]. Les patients ayant un score de Tokuhashi compris entre 12 et 15 points ont un bon pronostic avec une survie moyenne supérieure ou égale à 1 an; ceux ayant un score modéré (score compris entre 9 et 11 points) et un mauvais score (score de 0 à 8 points) ont une survie moyenne ne dépassant pas 6 mois.

**Tableau 1:** score de Tokuhashi

Critères	Points
**Etat général**	0
Médiocre	1
Moyen	2
Bon	3
**Déficit moteur (Frankel)**	-
Frankel B et B complet	0
Frankel C et D incomplet	1
Frankel E (aucun)	2
**Tumeur primitive**	-
Langue, estomac, vessie, œsophage, pancréas	0
Foie, vésicule biliaire, tumeur non identifiée	1
Autre	2
Rein, utérus	3
Thyroïde, seins, prostate, tumeur carcinoïde	4
**Nombre de métastases vertébrales**	-
>2	2
2	1
1	0
**Nombre de métastases osseuses extra-vertébrales**	-
>2	2
1-2	1
0	0
**Métastases viscérales**	-
Non résequable	2
Réséquable	1
Aucun	0
**Total**	**0 à 15**

**Analyse des données:** les données ont été analysées grâce aux logiciels Epi Info 7.2.2.6, Microsoft Word et SPSS (Statistical Package for the Social Sciences). Pour la corrélation entre la variable dépendante et les différentes variables indépendantes, nous avons fait une analyse de régression logistique multivariée. Ainsi, le test de Chi^2^ ou le test exact de FISHER ou le test de Wilcoxon a été utilisé pour rechercher une liaison entre les variables qualitatives. La courbe de Kaplan-Meier a été utilisée pour déterminer la survenue médiane chez les patients pendant l'analyse. Le seuil de significativité était de 5% ; la p-valeur <0,05 a été considérée comme statistiquement significative.

**Considérations éthiques:** nous avons obtenu l'accord verbal de tous les patients et/ou de leurs proches (personnes désignées comme personnes de confiance) pour participer à l'étude. L'identification des dossiers a été effectuée par numéro plutôt que par nom (en gardant l'anonymat du patient). Cette étude a été approuvée par le comité d'éthique et de recherche en santé (CNERS) par le décret … et les principes de la déclaration de Helsinki ont été respectés.

## Résultats

**Aspects épidémiologiques:** au total, 82 patients présentant des métastases osseuses ont été inclus, avec une prédominance masculine (53 hommes, soit 64,63%). L'âge moyen était de 65 ans chez les hommes (extrêmes allant de 59 à 70 ans) et de 58 ans chez les femmes (extrêmes allant de 50 à 65 ans). Concernant les comorbidités, l'hypertension artérielle était présente chez 18 hommes (34,0%) et 12 femmes (41,4%), tandis que le diabète sucré était retrouvé chez 4 hommes (7,5%) et 3 femmes (10,3%). Aucun patient n'était porteur de rétrovirose à VIH, ni de rhumatisme inflammatoire chronique (Tableau 2).

**Tableau 2:** caractéristiques sociodémographiques et cliniques

	Sexe				Total N = 8 21
	Masculin N = 531	Féminin N = 291	Ora*	IC à 95%	
**Age (année)**	65 (59-70)	58 (50-65)	1.25	0,98 - 1,02	64 (54-69)
**Délai d'évolution**	5 (3-11)	8 (5-13)	2.74	0,75 - 1,04	6 (3-12)
HTA*	18 (34,0)	12 (41,4)	0.36	0,45 - 1,15	30 (36,6)
**Diabète**	4 (7,5)	3 (10,3)	1.14	1,25 - 4,86	7 (8,5)
**Insuffisance renale**	2 (3,8)	2 (6,9)	0,54	0,98 - 2,93	4 (4,9)
**Insuffisance hépatique**	1 (1,9)	-	-	1,27 - 4,08	1 (1,2)
VIH**	-	-	-	-	-
RIC***	-	-	-	-	-
**Obésité**	-	-	-	-	-

Médiane (25%-75%) ; n (%); Ora: Odds Radio ajusté; HTA: Hypertension artérielle; VIH: Virus de l’Immunodéficience Humaine; RIC: Rhumatisme Inflammatoire Chronique (durée d’évolution en mois)

**Figure 1:**
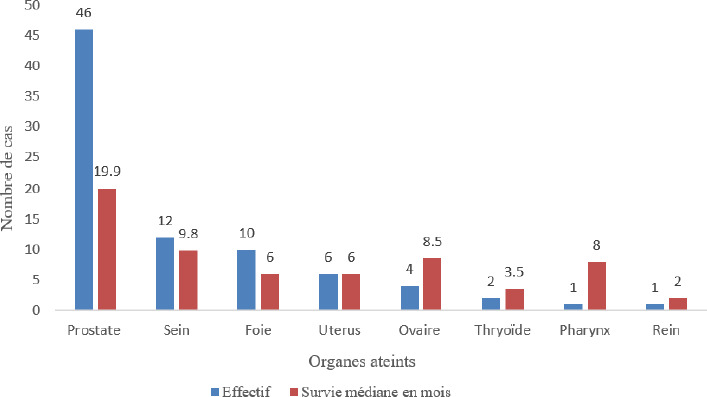
corrélation entre la survie médiane et le site primitif selon le score de Tokuhashi

**Caractéristiques cliniques et diagnostiques:** la durée d'évolution avant la prise en charge était en moyenne de 5 mois chez les hommes et 8 mois chez les femmes avec un intervalle de confiance (IC 95%: 0,75-1,04). La tumeur primitive la plus fréquemment retrouvée était le cancer de la prostate, observé chez 46 patients (56,09%), IC à 95%: 0,11-0,48 et HR : 0,23, suivi du cancer du sein chez 12 patients (14,63%), IC à 95%: 0,21-1,14 et HR: 0,48. D'autres localisations primitives étaient plus rares (Figure 1). La présence d'une image radiographique lytique (ORa: 3,87; IC à 95%: 1,66 - 4,85) et d'une image mixte (ORa: 2,58; IC à 95%: 1,38 - 4,74) a été le facteur statistique significatif de mauvais pronostic avec respectivement p<0,004 et p<0,001. (Tableau 3)

**Tableau 3:** relations entre la survie des patients et caractéristiques selon l'âge, les signes de la tumeur primitive, les signes imagériques

	Survie réelle (en mois)			Total N = 821			p-valeur2
	<6 N = 231	6-12 N = 281	>12 N = 311		IC à 95%	ORa*	
**Tranche d'âge (année)**							
[0,65[	14 (60,9)	16 (57,1)	13 (41,9)	43 (52,4)	0,98 - 1,02	2,56	0,321
[65,100[	9 (39,1)	12 (42,9)	18 (58,1)	39 (47,6)	0,34 - 0,86	4,23	
**Signes de tumeur primitive**	19 (82,6)	27 (96,4)	29 (93,5)	75 (91,5)	1,25 - 4,86	12,6	
VS* accélérée	23 (100)	24 (85,7)	28 (90,3)	75 (91,5)	1,27 - 4,08	7,63	0,218
**Image radiographique**							
Lytique	11 (47,8)	10 (35,7)	5 (16,1)	26 (31,7)	1,66 - 4,85	2,58	0,04
Condensante	6 (26,1)	12 (42,9)	22 (71,0)	40 (48,8)	--		
Mixte	6 (26,1)	6 (21,4)	4 (12,9)	16 (19,5)	1,38 - 4,74	3,87	0,01

N: effectif et (%): pourcentage; test du khi-deux d’indépendance; test exact de Fisher; vitesse de sédimentation; Ora: Odds Ratio ajusté

**Figure 2:**
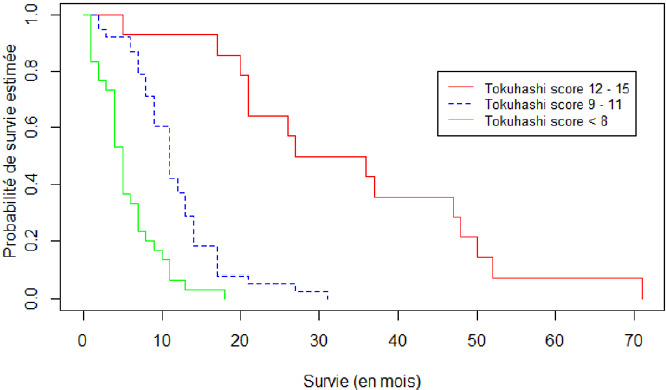
courbe de survie de Kaplan-Meier de fusion entre le score de Tokuhashi

**Aspects évolutifs et pronostiques:** il existait une corrélation statistiquement significative entre les prédictions pronostiques du score de Tokuhashi et la survie réelle des patients de notre échantillon. Des 14 patients qui avaient un bon pronostic selon le score de Tokuhashi, 92,9% ont survécu plus de 12 mois et 63,3% des patients qui avaient un mauvais pronostic ont survécu moins de 6 mois (ORa: 4,99; IC à 95%: 7,26 - 40,0) avec p = 0,001. (Tableau 4). La courbe de survie de Kaplan-Meier de chaque sous-groupe selon le score de Tokuhashi révèle que la probabilité de survie à 12 mois est très faible pour les patients de pronostic mauvais et modéré alors qu'elle est pratiquement de 90% pour les patients de bon pronostic (Figure 2). Une analyse multivariée entre les tumeurs les plus fréquentes et la survie selon le score de Tokuhashi a retrouvé que le cancer de la prostate (n = 46) était associé à une survie médiane réelle de 17,9 mois (Ora : 0,23; IC à 95%: 0,11-0,48); contre une survie prédite par le score de Tokuhashi de 9,87 mois. Le cancer du sein (n = 12) montrait une survie réelle de 9,87 mois (ORa: 0,48; IC à 95%: 0,21-1,14) avec une survie prédite de 8,92 mois (Tableau 5).

**Tableau 4:** répartition des patients selon le score de Tokuhashi et la survie réelle

Pronostic Tokuhashi	Survie réelle (en mois)			Total N = 821			p-valeur2
	<6 N = 231	6-12 N = 281	>12 N = 311		ORa*	IC à 95%	
Bon (Score: 12-15)	2 (8,7)	15 (53,6)	24 (77,4)	41 (50,0)	-		
Modéré (score: 9-11)	14 (60,9)	10 (35,7)	7 (22,6)	31 (37,8)	4.99	2,29 - 10,9	<0,001
Mauvais (score: 0-8)	7 (30,4)	3 (10,7)	0 (0,0)	10 (12,2)	17.0	7,26 - 40,0	<0,001

test exact de Fisher; ORa: Odd Ratio ajusté

**Tableau 5:** répartition des patients selon les types de cancer primaire, les scores et la survie réelle en mois

Site primaire	Effectifs	Survie réelle moyenne (mois)	IC* à 95%	ORa**	Score moyen de Tokuhashi modifié
Foie	10	6,0		-	5,40
Ovaire	4	8,5	0,19-1,95	0,60	7,25
Pharynx	1	8,0	1,10-6,33	0,80	9,00
Prostate	46	17,9	0,11-0,48	0,23	9,87
Rein	1	2,0	0,71-54,2	6,19	8,00
Sein	12	89,8	0,21-1,14	0,48	8,82
Thyroïde	2	3,5	0,68-16,4	3,33	7,50
Utérus	2	6,0	0,41-3,16	1,14	7,50
**Total général**	**82**	**13,3**	**-**	**-**	**8,79**

IC: Intervalle de confiance; ORa: Odds Ratio ajusté

## Discussion

Nous avons mené une étude rétrospective de type transversal, descriptive et analytique dans les services de rhumatologie du Togo, dont l'objectif général était d'évaluer la survie des patients souffrant de métastases vertébrales en milieu rhumatologique togolais. Dans notre étude, la tumeur primitive la plus fréquemment retrouvée était le cancer de la prostate, observé chez 46 patients (56,09%), suivie du cancer du sein chez 12 patients (14,63%). L'ostéolyse était statistiquement associée à un mauvais pronostic. Le cancer de la prostate était associé à une survie réelle de 17,9 mois contre une survie de 9,87 mois prédite selon le score de Tokuhashi.

**Aspects épidémiologiques:** les données épidémiologiques des métastases osseuses en Afrique noire ne sont pas fiables, car la plupart des études sont hospitalières et sont réalisées dans un délai très limité. Mais des similitudes sont rapportées concernant leurs incidences qui varient d'un pays à l'autre et leur survenue chez des patients jeunes, comme rapporté dans notre étude, qui est de 64 ans. Dans certaines séries africaines, la moyenne d'âge au moment du diagnostic est de 55,9 ans au Cameroun, de 55 ans au Togo et de 46,4 ans au Mali [3,4]. La prédominance masculine rapportée dans notre étude (53 hommes, soit 63,85%) a été rapportée dans la littérature africaine et dans certaines études occidentales [4,5]. Nos données sont contraires à celles de Bouthors *et al*. qui, dans leur étude, ont rapporté une prédominance féminine (54%) et un âge moyen de diagnostic de 62,2 ± 12,5 ans pour les femmes et de 66,4 ± 11,5 ans chez les hommes [10]. La relative jeunesse de la population africaine et la tradithérapie souvent recommandée dans notre contexte expliqueraient ces résultats. La prédominance du cancer de la prostate qui est l'une des tumeurs solides les plus fréquentes et le degré d'exposition à certains facteurs de risque dans la vie courante qui sont pourvoyeurs de cancers expliqueraient en partie ces données.

**Caractéristiques cliniques et diagnostiques:** dans notre étude, aucune différence statistiquement significative n'avait été trouvée entre les tranches d'âge (IC à 95%: 0,98 - 1,02) et la tumeur primitive. Arrigo *et al*. dans leur étude observent que l'âge supérieur ou égal à 70 ans serait associé à la survie des métastases osseuses et proposent même de l'inclure dans le score de Tokuhashi comme une valeur indépendante [11]. En 1996, Tatsui *et al*. soulignaient dans leur étude que la survie à 1 an des patients atteints de métastases osseuses était fortement liée à la tumeur primitive avec des proportions variables (83% pour le cancer de la prostate, 77% pour le sein, 51,2% pour le rein et 21,7% pour le poumon) [12]. Même si aucun lien n'a été mis en évidence entre le foyer primitif et la survie réelle des patients selon le score prédictif de Tokuhashi, il faut souligner que Haman *et al*. ont observé une forte prédominance du cancer de la prostate (41,6%) suivie du cancer du sein (23%) [13]. Ce même constat avait été fait dans notre étude, avec un lien statistiquement significatif entre le site primitif de la tumeur et la survie médiane des patients. En Afrique subsaharienne, le cancer de la prostate et celui de la femme sont les cancers solides les plus fréquemment observés. L'absence de registre de cancer dans notre contexte, le recours fréquent de nos populations à la médecine traditionnelle et l'insuffisance du plateau technique avec le manque de disponibilité des outils informatiques de suivi des patients pourraient expliquer cette différence de résultat. Par contre, le type d'image radiologique retrouvée avait un impact sur la survie des patients avec une différence statistiquement significative avec un taux de décès plus élevé en moins de 6 mois observé chez les patients avec des images lytiques. Au Cameroun, Haman *et al*. ont retrouvé dans leur étude aussi une prédominance des lésions lytiques [13]. Les complications métaboliques de lyse osseuse (l'hypercalcémie) et les atteintes neurologiques à type de compression médullaire par recul du mur postérieur seraient à l'origine de ces phénomènes.

**Aspects évolutifs et pronostiques:** la survie variait selon le groupe reparti en fonction du score du Tokuhasi. Sept patients (30,4%) survivaient en moyenne 6 mois (score de Tokuhashi 8 - 10 points), 3 patients (10,7%) avaient vécu entre 6 et 12 mois et aucun présentant un mauvais score n'avait vécu au-delà de 12 mois dans notre étude. Une survie supérieure ou égale à 12 ans avait été observée dans le groupe des patients présentant un bon score de Tokuhashi (score de 2 à 4 points). Dans son étude, Eap *et al*. rapportent des données similaires avec une répartition des patients dans les trois groupes pronostiques définis par le score de Tokuhashi semblable, et qui était la suivante: score 0 à 8 (survie prédite < 6 mois), 105 patients (40,4%); score 9 à 11 (survie prédite > 6 mois), 82 patients (31,5%); et score 12 à 15 (survie prédite > 12 mois), 73 patients (28,1%) [14]. Au Cameroun, Haman *et al*. avaient observé que seuls 19 patients (18,81%) avec un bon score de Tokuhashi survivaient au-delà de 12 mois, et 41 patients (40,59%) survivaient dans moins de 6 mois [13]. La moyenne de la survie réelle dans notre étude était de 13,3 mois avec une survie moyenne de 8,79 mois pour celui de Tokuhashi modifié. La concordance de nos résultats avec ceux des autres vient montrer l'intérêt de l'application de ce score chez tous les patients souffrant de métastases osseuses afin de limiter l'acharnement thérapeutique dont sont souvent victimes nos patients dans nos pays où les moyens économiques sont limités.

Les métastases osseuses sont révélatrices de certains cancers ostéophiles et leur incidence varie en fonction de l'organe en question. Ces métastases nécessitent une prise en charge spécialisée, en particulier chirurgicale, pour le traitement des complications neurologiques (compression médullaire ou syndrome de la queue de cheval) qui peuvent engager le pronostic vital du patient. Dans notre contexte de pratique rhumatologique, l'insuffisance du plateau technique (bilan d'extension tumorale non accessible à certains de nos patients par manque de moyens financiers), la notion disponibilité de certaines thérapeutiques ciblées spécifiques de certaines métastases telles que les biothérapies, certaines hormonothérapies, la chirurgie dont le coût élevé et l'absence de spécialiste) ont été les limites rencontrées dans notre étude. Le nombre d'atteintes a été jugé selon les résultats de la radiographie et/ou du scanner qui ont été réalisés chez certains patients. D'autres examens plus exhaustifs et plus sensibles comme une IRM du rachis entier, la scintigraphie osseuse pour détecter les métastases osseuses et/ou viscérales n'ont pas été réalisés du fait de leur indisponibilité ou de leur coût extrêmement élevé. Les patients de notre cohorte n'ont bénéficié que de traitements très limités associant des antalgiques prescrits selon l'intensité de la douleur, la corticothérapie pour certains d'entre eux en l'absence de contre-indications, les biphosphonates (acide zolédronique 5 mg/4 ml) en perfusion mensuelle et de la kinésithérapie pour les patients présentant déjà des complications neurologiques. Malgré ces biais de suivi recensés, cette étude garde tout son intérêt dans l'évaluation du pronostic vital chez les patients souffrant de métastases osseuses dans notre contexte.

## Conclusion

Les métastases osseuses sont fréquentes au cours de nombreux cancers ostéophiles, en particulier le cancer de la prostate, du sein et du poumon. L'ostéolyse était statistiquement associée à un mauvais pronostic. Le cancer de la prostate était associé à une survie réelle de 17,9 mois contre une survie de 9,87 mois prédite selon le score de Tokuhashi. Le cancer de la prostate (Ora: 0,23; IC à 95%: 0,11-0,48) et celui du sein (ORa: 0,48; IC à 95%: 0,21-1,14) sont les cancers les fréquents et statiquement associés à la survie des patients selon le score de Tokuhashi avec une survie réelle de 17,9 mois contre une survie prédite par le score de Tokuhashi de 9,87 mois et cancer du sein avec une survie réelle de 9,87 mois contre une survie prédite de 8,82. Le score de Tokuhashi modifié est un outil pronostique qui permet de prédire la survie des patients atteints de métastases osseuses et son utilisation doit être incorporée dans nos pratiques quotidiennes pour optimiser la prise en charge des patients.

### Etat des connaissances sur le sujet

- La survie des patients dépend du type de tumeur primitive;
- L'état général influence l'évolution et la survie des patients souffrant de tumeur osseuse secondaire;
- Le score de Tokuhashi est un score validé et connu, utilisé surtout en chirurgie oncologique.

### Contribution de notre étude à la connaissance

- Apports sur les connaissances épidémiologiques des métastases osseuses en Afrique subsaharienne, applicabilité effective du score de Tokuhashi dans nos milieux à ressources limitées (médecine nucléaire manquante);
- Les facteurs statistiquement associés au mauvais pronostic étaient la présence d'une image radiographique lytique (ORa: 3,87; IC à 95%: 1,66 - 4,85) et d'une image mixte (ORa: 2,58; IC à 95%: 1,38 - 4,74) avec respectivement p<0,004 et p<0,001;
- Cette étude révèle que la survie réelle des patients atteints de cancers fréquents est globalement supérieure à celle prédite par le score de Tokuhashi, malgré un taux de survie très faible pour les patients présentant un mauvais pronostic initial.

## Remerciements

Nos remerciements vont à l'endroit de tous les maîtres, des collègues et des autorités administratives des différents services de Rhumatologie (CHU Kara, CHU Sylvanus Olympio et l'hôpital de Bè) où cette étude a été réalisée. Aussi, nous remercions les patients qui ont accepté de participer volontairement à l'étude.
